# Novel Roles for Staufen1 in Embryonal and Alveolar Rhabdomyosarcoma via c-myc-dependent and -independent events

**DOI:** 10.1038/srep42342

**Published:** 2017-02-17

**Authors:** Tara E. Crawford Parks, Kristen A. Marcellus, Jonathan Langill, Aymeric Ravel-Chapuis, Jean Michaud, Kyle N. Cowan, Jocelyn Côté, Bernard J. Jasmin

**Affiliations:** 1 Department of Cellular and Molecular Medicine, Faculty of Medicine, University of Ottawa, Ottawa, Ontario, Canada; 2 Centre for Neuromuscular Disease, Ottawa, Ontario, Canada; 3 Department of Pathology and Laboratory Medicine, University of Ottawa, The Ottawa Hospital and Children’s Hospital of Eastern Ontario, Ottawa, Ontario, Canada; 4 Molecular Biomedicine Program, Children’s Hospital of Eastern Ontario Research Institute, Ottawa, ON, Canada; 5 Department of Surgery, Children’s Hospital of Eastern Ontario, University of Ottawa, Ottawa, Ontario, Canada

## Abstract

Rhabdomyosarcoma is the most common soft tissue sarcoma in children and young adults. Rhabdomyosarcomas are skeletal muscle-like tumours that typically arise in muscle beds, and express key myogenic regulatory factors. However, their developmental program remains blocked in the proliferative phase with cells unable to exit the cell cycle to fuse into myotubes. Recently, we uncovered a key role for the RNA-binding protein Staufen1 during myogenic differentiation through the regulation of c-myc translation. Given the known implication of c-myc in rhabdomyosarcoma, we hypothesized in the current work that Staufen1 controls rhabdomyosarcoma tumorigenesis. Here, we report for the first time the novel role of Staufen1 in cancer, specifically in rhabdomyosarcoma. We demonstrate that Staufen1 is markedly upregulated in human rhabdomyosarcoma tumours and cell lines as compared to normal skeletal muscle. Moreover, we show that Staufen1 promotes the tumorigenesis of embryonal and alveolar rhabdomyosarcoma subtypes both in cell culture and in animal models. Finally, our data demonstrate that Staufen1 has differential roles in embryonal versus alveolar rhabdomyosarcoma through the control of proliferative and apoptotic pathways, respectively. Together, these results provide the first evidence for Staufen1’s direct implication in cancer biology. Accordingly, Staufen1 thus represents a novel target for the development of future therapeutic strategies for rhabdomyosarcoma.

Rhabdomyosarcoma (RMS) is the most common soft tissue sarcoma in children and young adults^1^. RMS cases account for approximately 50% of all pediatric soft tissue sarcomas, and 8% of all pediatric neoplasms^2^. The World Health Organization’s classification for tumours of soft tissue and bone subdivides RMS into four subtypes: embryonal (ERMS), alveolar (ARMS), pleomorphic, and spindle cell/sclerosing RMS, each with distinct genetic, histological and clinical features^3^. The two major forms of RMS are ERMS and ARMS with 2/3 of all RMS cases diagnosed as ERMS. ERMS is most prevalent in children less than 10 years of age. This subtype is genetically heterogeneous with the activation of several oncogenic signaling pathways in combination with the loss of tumour surveillance mechanisms. Although a single mutation for all ERMS cases is not described, many are a result of the loss in heterozygosity at chromosome 11p15.5^4^. In contrast, ARMS tumours are commonly found in children as well as young adults. This subtype is often a result of chromosomal translocations t(2;13)(q35;q14) or t(1;13)(q36;q14), which account for approximately 60% or 20% of ARMS cases, respectively. These translocations cause the fusion between the paired box (*PAX*) genes, *PAX3* or *PAX7* and the 3′end of the Forkhead box O1 (*FOXO1*) locus located on chromosome 13^5^,^6^,^7^. The production of the PAX3-FOXO1 or PAX7-FOXO1 fusion protein is oncogenic and drives the formation of ARMS tumours. However, approximately 20% of ARMS tumours are fusion negative despite exhibiting the classic alveolar phenotype^8^,^9^,^10^. This makes it difficult to determine whether ARMS represents multiple clinical and biological entities sharing a common phenotype.

Modern therapeutic regimens combining the use of surgery, radiation therapy and chemotherapy have improved the overall 5-year survival rate. Nonetheless, the 5-year survival rate for ERMS and ARMS remains at approximately 67% and 49%, respectively^11^. Moreover, amongst RMS cases in which the tumour is metastatic upon diagnosis, 3-year event-free survival rates are just over 30%, being significantly and adversely influenced by alveolar histology^12^. The treatment plan for metastatic RMS has not improved for several decades and, consequently, there is a clear need to better define the molecular mechanisms driving RMS pathogenesis in an attempt to identify novel and relevant therapeutic targets.

RMS are generally thought of as skeletal muscle-like tumours since they typically arise in muscle beds and express key myogenic regulatory factors (MRFs)^13^. Although RMS cells express MRFs, their developmental program remains blocked in the proliferative phase and the cells are unable to exit the cell cycle to fuse into multi-nucleated myotubes and mature myofibers^14^. The Ras/MEK/ERK signaling pathway is known to be highly activated in ERMS leading to an accumulation of c-myc^15^. The increased expression of c-myc is proto-oncogenic since c-myc dimerizes with its binding partner Max to drive expression of oncogenic target genes causing cancer cells to proliferate indefinitely^16^. The inhibition of c-myc via the MEK/ERK inhibitor U0126 in ERMS cells promotes myogenic differentiation resulting in the formation of terminally differentiated muscle-like cells^17^. In addition, the down-regulation of c-myc in ERMS xenografts significantly reduced tumour growth^18^ and enhanced radiosensitivity of ERMS tumours^19^. More recently, it was described that c-myc drives ERMS transformation and radioresistance by promoting DNA repair^20^. Although c-myc expression has not been a primary focus in ARMS, it has also been reported to be amplified in some ARMS cell lines and tumours. In fact, early studies suggested that c-myc expression may be higher in ARMS compared to ERMS^21^. Such pathways involved in maintaining the proliferative capacity of cells, may thus represent ideal targets for therapeutic intervention focused on rescuing the impaired differentiation program thereby alleviating the malignant characteristics of these cells.

As part of our efforts focused on the role of RNA-binding proteins (RBPs) in neuromuscular biology and on their therapeutic potential for various neuromuscular disorders, we became interested several years ago in examining the impact of Staufen1 in skeletal muscle development, at the mature neuromuscular junction, as well as in Myotonic Dystrophy Type I (DM1)^22^,^23^,^24^,^25^,^26^. Staufen1 is a multi-functional RBP with several key roles in mRNA localization^27^, stability^28^,^29^,^30^, translation^22^,^31^,^32^,^33^, the cell cycle^34^ and alternative splicing^23^,^25^,^33^. There are two protein isoforms of Staufen1 generated through alternative splicing of its pre-mRNA, Stau1-55 and Stau1-63, which are ubiquitously expressed^35^,^36^. In this context, we recently described a key functional role of Stau1-55 in myogenic differentiation of both mouse and human skeletal muscle^22^. Our findings showed that Stau1-55 levels decreased progressively during differentiation of myogenic cells and during embryonic muscle development. Such a reduction in Stau1-55 expression is necessary for proliferating myoblasts to exit the cell cycle and differentiate^22^. Mechanistically, we uncovered that Stau1-55 overexpression impairs myogenic differentiation by increasing the translation of c-myc, a factor known to delay cell-cycle withdrawal and terminal differentiation of skeletal muscle cells^22^,^37^.

Given the impact of c-myc in the RMS pathology^15^,^16^,^17^,^18^,^19^,^20^,^38^, and our recent findings showing that Staufen1 regulates c-myc during skeletal muscle differentiation^22^, we hypothesize in the current study that Staufen1 controls RMS tumorigenicity. To this end, we performed a series of complementary experiments to examine the impact of Staufen1 in RMS tumorigenesis. Our findings demonstrate that Staufen1 is markedly elevated in ERMS and ARMS. Moreover, we show that overexpression of Staufen1 in RMS promotes tumorigenesis of both subtypes in cell culture and xenograft models. However, Staufen1 appears to assume differential roles in ERMS versus ARMS through the regulation of proliferative and apoptotic mechanisms, respectively. Collectively, these results show for the first time the direct involvement of Staufen1 in cancer biology thereby highlighting its therapeutic potential as a novel target.

## Results

### Staufen1 is increased in Human Embryonal and Alveolar Rhabdomyosarcoma

**Figure 1:**
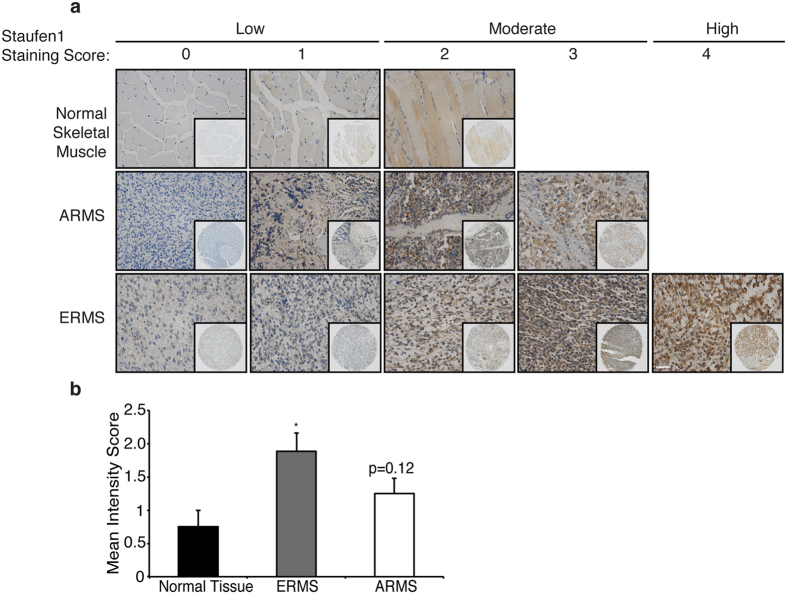
Staufen1 is overexpressed in human rhabdomyosarcoma. Tissue microarrays were used to evaluate Staufen1 expression by immunohistochemistry in human normal skeletal muscle and primary rhabdomyosarcoma core biopsies. (**a**) Intensity scoring of tissues was performed and analyzed using a 4-point scoring system with 0–1 = low expression, 2–3 = moderate expression and 4 = high expression. Representative images of scored Staufen1 staining in normal skeletal muscle tissue (n = 8), Embryonal Rhabdomyosarcoma (ERMS) (n = 26), and Alveolar Rhabdomyosarcoma (ARMS) (n = 24) using anti-Staufen1 antibodies (brown) and hematoxylin stain for nuclei (blue); 20X objective, scale bar = 50 μm. Insert represents lower magnification images at 5X magnification to visualize total core biopsy sample. (**b**) Quantification of the mean intensity score, data are Mean ± SEM, *P < 0.05.

To investigate Staufen1 expression in RMS, immunohistochemistry was first performed on a commercially available, high-density tissue microarray. We assessed a total of 26 ERMS, and 24 ARMS tumours as well as 8 normal skeletal muscle samples for Staufen1 expression. Since RMS tumours are thought to originate from a muscle-like cell lineage, skeletal muscle samples are routinely used as controls for the comparison of gene expression in RMS^13^. In these experiments, we performed scoring for Staufen1 staining using a 4-point scale where 0–1 is low, 2–3 is moderate, and 4 represents high Staufen1 expression (see additional details in Methods). Representative images are shown in Fig. 1a and the mean intensity score was calculated for each tissue (Fig. 1b). Analyses revealed an increase in Staufen1 staining intensity in ERMS (P < 0.05) and a trend towards increased expression in ARMS tumours (P = 0.12) as compared to normal muscle tissue (Fig. 1b). It is important to note that in the ARMS samples examined, patient age, treatment history, tumour location and the PAX3/7-FOXO1 status likely contributed to the lack of significance observed in Staufen1 expression. Detailed examination of intensity score distributions demonstrated that a higher percentage of RMS tumours expressed Staufen1 at moderate and high levels (score >2) as compared to normal muscle (Table 1). In fact, 87.5% of normal skeletal muscle tissue had low and 12.5% had moderate expression of Staufen1, consistent with our previous analysis of Staufen1 levels in mouse adult skeletal muscle^22^. In contrast, 46.2%, 34.6% and 19.2% of ERMS tumours expressed Staufen1 at low, moderate, or high levels, respectively (Table 1). In addition, 66.7% and 33.3% of ARMS tumours expressed Staufen1 at low and moderate levels, respectively (Table 1). In all of the samples, the localization of Staufen1 was predominantly cytoplasmic with a lower level of expression seen in nuclei. While no low-grade tumours (grade I or II) were present in the TMA, the majority of the tumour samples were grade III (n = 47) and very few were classified as grade IV (n = 3). As a result, correlation of Staufen1 expression across tumour grade was not possible with this sample set.

Next, using an *in vitro* cell culture system, we examined Staufen1 expression in human primary Skeletal Muscle Cells (SkMC), ERMS (RD) and ARMS (RH30) cells. RD cells are one of the most commonly used ERMS cell line. These cells were developed from a biopsy of pelvic ERMS previously treated with cyclophosphamide and radiation, and they were found to be resistant to treatment^39^. RD cells have 51-hyperdiploid chromosomes and contain several amplifications and mutations in cancer-related genes such as *MYC* amplification^40^, *NRAS* mutation (Q61H)^38^, and homozygous mutation of *TP53*^41^,^42^. The RH30 cell line was derived from a bone marrow sample of a 16-year-old male with untreated metastatic ARMS^43^. These cells express the t(1;13) translocation and display near-triploid chromosomes^44^, amplification of the chromosomal region involving *CDK4*^45^,^46^,^47^, and mutated *TP53*^41^,^42^,^44^.

**Figure 2:**
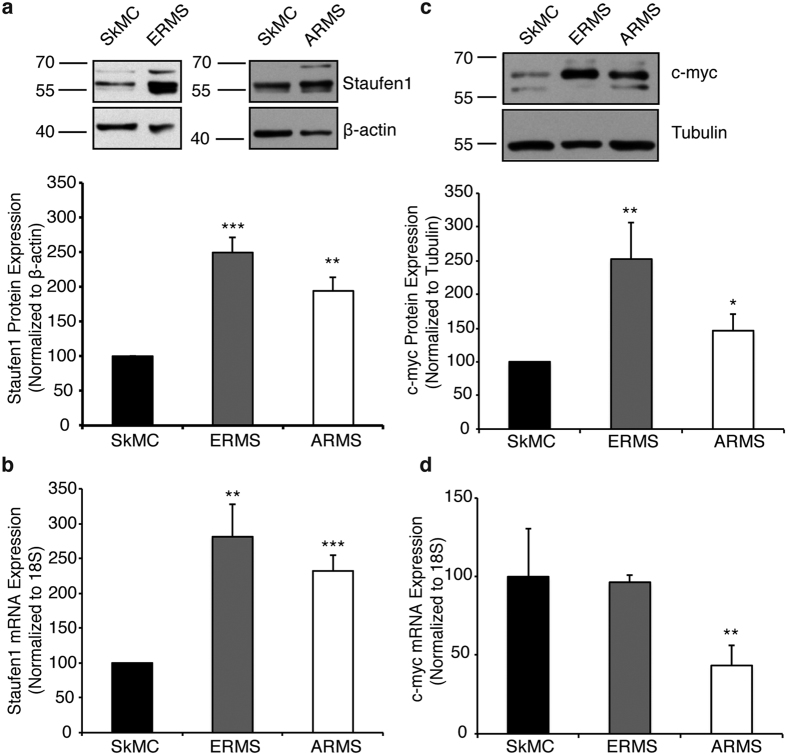
Staufen1 and c-myc are overexpressed in ERMS and ARMS cell lines. Expression of Staufen1 and c-myc were determined in human skeletal muscle cells (SkMC), ERMS (RD) and ARMS (RH30) cells. (**a**) Western blot using anti-Staufen1 antibodies and β-actin as a loading control. The predominant Stau1-55 isoform was quantified (n = 4). (**b**) qRT-PCR using primers specific for Staufen1 mRNAs and normalized to total levels of 18 S rRNA (n = 6). (**c**) Western blot using anti-c-myc antibodies and tubulin as a loading control (n = 5). (**d**) qRT-PCR using primers specific for c-myc mRNAs and normalized to total levels of 18 S rRNA (n = 5). All quantifications are represented as a percentage relative to SkMC (n = 5). Data are mean ± SEM, *P < 0.05, **P < 0.01, ***P < 0.001.

Western blot and quantitative RT-PCR (qRT-PCR) were performed to determine Staufen1 protein and mRNA expression, respectively, in ERMS and ARMS cells as compared to SkMCs (Fig. 2a,b). Results showed that both Stau1-55 and Stau1-63 isoforms were increased at the protein level in ERMS and ARMS with Stau1-55 being the predominant isoform. Quantification of Stau1-55 revealed an ~150% and ~95% increase, respectively (P < 0.01, Fig. 2a). Staufen1 mRNA levels were also increased by ~180% and ~132% in ERMS and ARMS cells, respectively (P < 0.01, Fig. 2b). In these same cells, we observed an increase in c-myc, a key mediator of RMS^15^,^18^,^19^,^38^. C-myc protein expression was augmented by ~152% and ~46% in ERMS and ARMS cells, respectively (P < 0.01, Fig. 2c). In contrast to the increase in c-myc protein levels, no change in c-myc mRNA expression was detected in ERMS (P > 0.05) whereas a decrease was seen in ARMS (~60% decrease; P < 0.05) (Fig. 2d). These data are in complete agreement with our recent demonstration that Staufen1 regulates translation of c-myc^22^, thereby maintaining RMS cells in a proliferative state and promoting their tumorigenesis.

These results were confirmed by western blot for Staufen1 expression in human ERMS and ARMS tumours with ~325% and ~650% increase in Staufen1 levels, respectively, as compared to adult skeletal muscle (Figure S1a). In addition, c-myc protein expression was also increased in human primary tumour samples with an ~727% and ~321% increase in ERMS and ARMS, respectively, as compared to adult skeletal muscle (Figure S1b). Interestingly, Staufen1 and c-myc levels in RMS tumours appeared similar to those observed in fetal skeletal muscle (Figure S1a,b). To demonstrate that the increase of Staufen1 and c-myc in the RD and RH30 cell lines are representative of ERMS and ARMS, we further examined their expression across a panel of RMS cell lines. Our data showed that Staufen1 and c-myc are elevated in the ERMS cell lines RH36 and RH18 as compared to control Human Skeletal Muscle Myoblasts (HSMM) (Figure S1c,d). Similarly, Staufen1 and c-myc are increased in the ARMS cell line RH41, consistent with the observed expression in RH30 cells as compared to control cells (Figure S1c,d). Most striking is the strong correlation between Staufen1 and c-myc across the cell lines examined denoted by a Pearson correlation coefficient of 0.96 (P < 0.01). Together, these data strengthen the conclusion that Staufen1 is increased in ERMS and ARMS.

### Staufen1 Enhances the Tumorigenic Potential of RMS cells

To assess the impact of Staufen1 in RMS cells, we used a mix of three independent Staufen1 targeting shRNAs to knockdown Staufen1 in ERMS and ARMS cell lines. Several attempts were made to develop cell lines stably expressing Staufen1-shRNA via antibiotic selection, however these cells were not viable. Therefore, control (CTL) non-targeting shRNA or Staufen1-targeting shRNA lentiviruses were used to transduce RMS cell lines to achieve a Staufen1 knockdown. To confirm efficient Staufen1 knockdown in ERMS and ARMS cells, we performed immunofluorescent staining using anti-Staufen1 antibodies. Results showed a consistent knockdown across the entire cell population (Figure S2). In addition, these cells were probed for the terminal differentiation marker Myosin Heavy Chain (MHC) to determine if Staufen1 knockdown induced precocious differentiation as previously demonstrated in C2C12 cells^48^. However, ERMS and ARMS expressing Staufen1-shRNA did not express MHC (data not shown).

**Figure 3:**
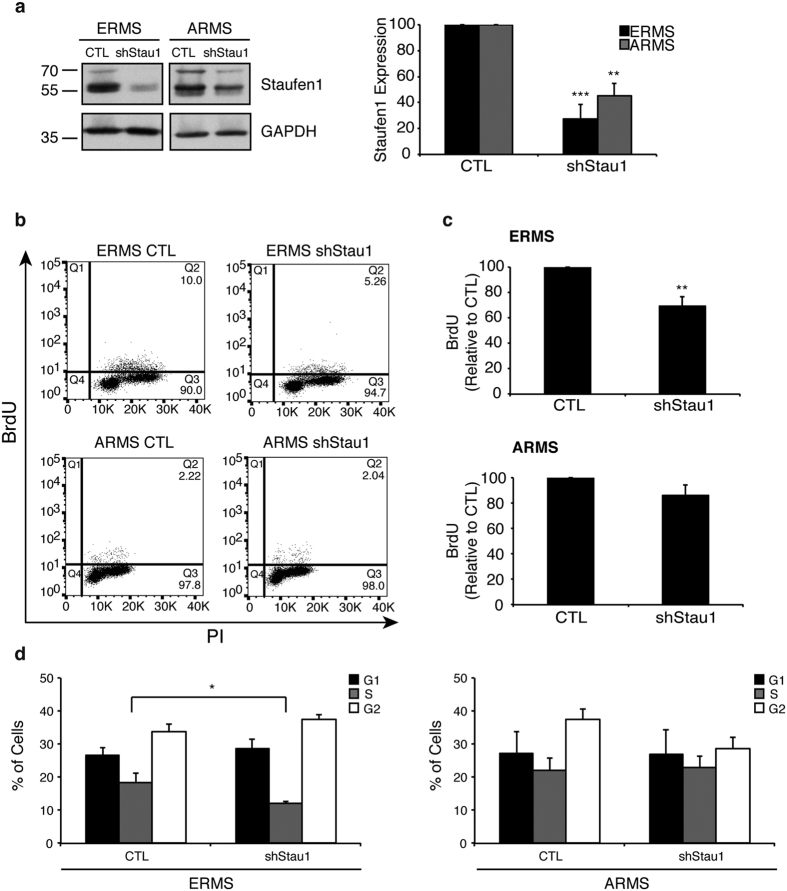
Staufen1 regulates the proliferation and cell cycle of ERMS cells. Analysis of cell proliferation was performed after 72 hours of Control (CTL) or Staufen1-shRNA expression (shStau1) in ERMS and ARMS cells. (**a**) Representative western blot of Staufen1 expression with GAPDH as a loading control in ERMS (RD) and ARMS (RH30) cells. Quantification of n = 4 and n = 3, respectively, is represented as a percentage of the CTL. (**b**) Proliferation was assessed by two-parameter flow cytometry and representative dot plots following 2 h of BrdU incorporation and Propidium Iodide (PI) staining in ERMS (RD) and ARMS (RH30) cells expressing CTL or Staufen1-shRNAs. (**c**) Quantification of BrdU incorporation is represented as a percentage relative to CTL in ERMS (RD) and ARMS (RH30) cells, n = 4 and n = 3, respectively. (**d**) Cell cycle analysis was examined by flow cytometry and the percentage of the cells in G1, S and G2 phases are indicated. Data are Mean ± SEM, *P < 0.05, **P < 0.01, ***P < 0.001.

Western blot analysis confirmed Staufen1 knockdown in both cell lines at 72 hours post-infection (Fig. 3a). First, we evaluated cell proliferation using flow cytometry to measure bromodeoxyuridine (BrdU) and propidium iodide (PI) staining. Following Staufen1 knockdown, ERMS cells showed an ~30% decrease in BrdU incorporation, which revealed their lower proliferative capacity (P < 0.01) as compared to CTL (Fig. 3b,c). By contrast, ARMS cell proliferation was unaffected (Fig. 3b,c). Cell cycle analysis was performed on these cells and in agreement with the BrdU incorporation experiments, there was a decrease in the percentage of cells in S-phase (P < 0.05, Fig. 3d).

**Figure 4:**
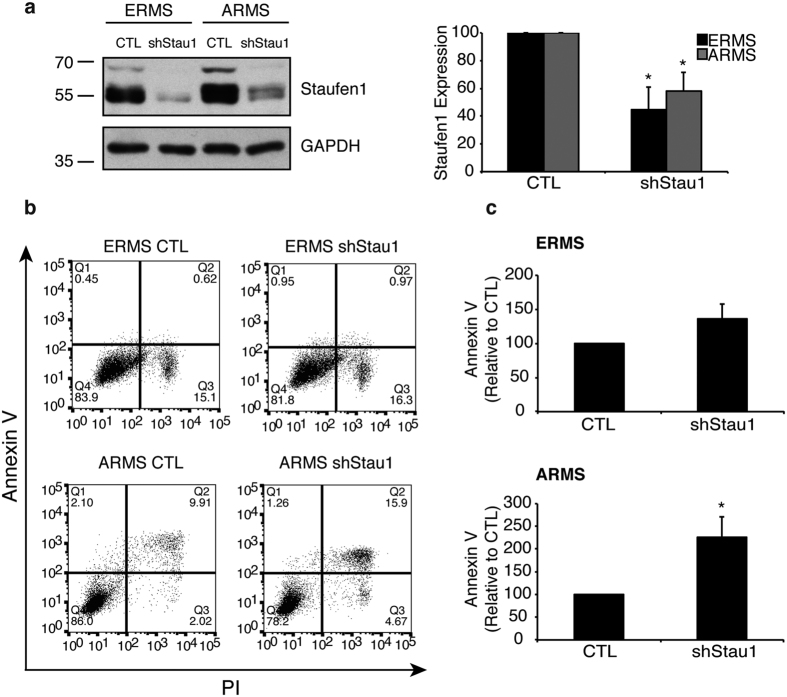
Staufen1 regulates apoptosis in ARMS cells. Analysis of apoptosis was performed after 72 hours of Control (CTL) or Staufen1-shRNA expression (shStau1) in ERMS and ARMS cells. (**a**) Representative western blot of Staufen1 expression with GAPDH as a loading control in ERMS (RD) and ARMS (RH30) cells. Quantification of n = 3 is represented as a percentage of the CTL. (**b**) Apoptosis was assessed by two-parameter flow cytometry and representative dot plots of CTL and Staufen1-shRNA expressing ERMS (RD) and ARMS (RH30) cells stained with Annexin V and Propidium Iodide (PI). (**c**) Quantification of Annexin V staining is represented as a percentage relative to CTL (n = 3). Data are Mean ± SEM, *P < 0.05.

Next, dual staining of cells with Annexin V and PI was performed and analyzed by flow cytometry to assess apoptosis in RMS cells expressing Staufen1-shRNA. Western blot analysis demonstrated reduced Staufen1 levels in both cell lines (Fig. 4a). Our data showed that apoptosis in ERMS cells was unaffected. However, ARMS cells showed a significant increase in Annexin V staining (~126%, P < 0.05) suggesting that Staufen1 protects ARMS cells from apoptosis (Fig. 4b,c). These data indicate that Staufen1 has different roles in regulating ERMS and ARMS tumorigenesis.

**Figure 5:**
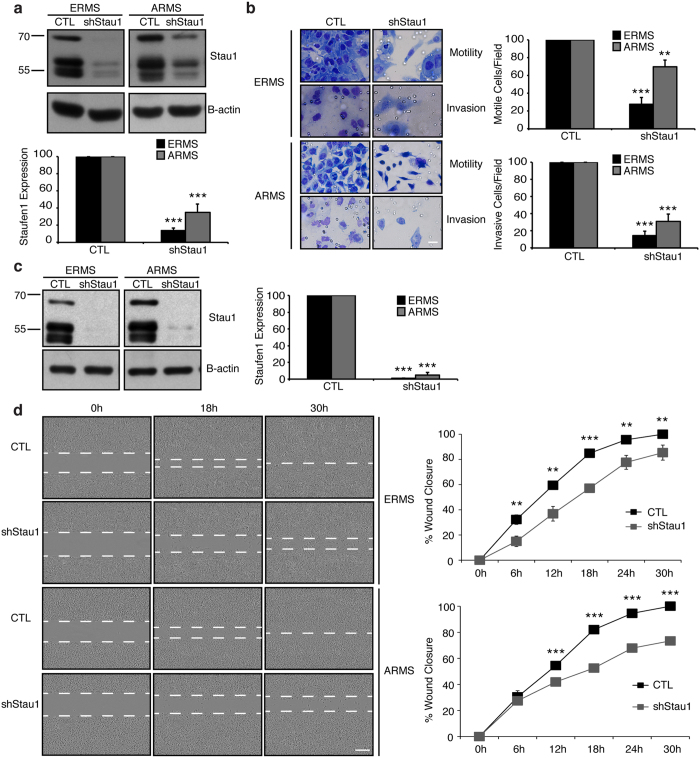
Staufen1 knockdown inhibits RMS cell invasion and migration. Motility and Invasion assays were performed following 48 hours (h) of Control (CTL) or Staufen1-shRNA (shStau1) expression. (**a**) Western blot analysis of Staufen1 expression showing a representative blot of Staufen1 with β-actin as a loading control in ERMS (RD) and ARMS (RH30) cells. (**b**) Cells were seeded into transwell chambers containing membranes coated with or without Matrigel and incubated for 72 h and 48 h for ERMS (RD) and ARMS (RH30) cells, respectively. Representative images of cells that passed through the transwell chamber at 40X magnification, scale bar = 20 μm, are displayed. Average number of cells/field of view is quantified for cell motility (no matrigel) and invasion (matrigel) as a percentage relative to CTL. Migration assays were performed after 48 h of Staufen1 knockdown and (**c**) is a representative western blot and quantification of Staufen1 expression with β-actin as a loading control in ERMS (RD) and ARMS (RH30) cells. (**d**) Cells were seeded into culture insert wells for 24 h, following removal of insert, a 500 μm cell free gap represents 0 h, and consecutive photos were taken at 6 h intervals for a total of 30 h. Representative images at 10X magnification, scale bar = 300 μm. Quantification of the gap is represented as % wound closure normalized to cell confluency. Quantifications are for all data are n = 3. Data are Mean ± SEM, **P < 0.01, ***P < 0.001.

To determine the role of Staufen1 on the metastatic potential of RMS, we investigated the impact of Staufen1 knockdown on the invasion and migration properties of these cells. Western blotting confirmed an ~85% and ~65% reduction of Staufen1 in ERMS and ARMS cells, respectively (Fig. 5a). ERMS and ARMS cells were plated into transwell chambers containing a porous membrane coated with or without matrigel. In the absence of matrigel, the knockdown of Staufen1 decreased both ERMS and ARMS cell motility by ~70% and ~30%, respectively (P < 0.01, Fig. 5b). Furthermore, we assessed the invasion of cells in the presence of matrigel and observed that both ERMS and ARMS cell invasion was decreased by ~85% and ~70%, respectively (P < 0.001, Fig. 5b). The facts that both cell motility and invasion were decreased in ERMS and ARMS cells with Staufen1 knockdown support the idea that Staufen1 has a dual role in these RMS subtypes.

Next, we performed migration assays to further evaluate the impact of Staufen1 on RMS cell motility and migration. Western blotting confirmed Staufen1 knockdown of ~100% efficiency in both cell lines (Fig. 5c). Cells were seeded into culture inserts for 24 hours and then a 500 μm cell free gap was created by removing the insert. Removal of the insert represented time 0 hour. Migration of the cells was subsequently evaluated at 6 hour-intervals for a total of 30 hours. Results of these experiments revealed that upon Staufen1 knockdown, ERMS and ARMS cells showed significant reductions in migration at almost all time points as compared to CTL cells (Fig. 5d).

### Decreasing Staufen1 Expression Inhibits RMS Tumour Growth *in vivo*

**Figure 6:**
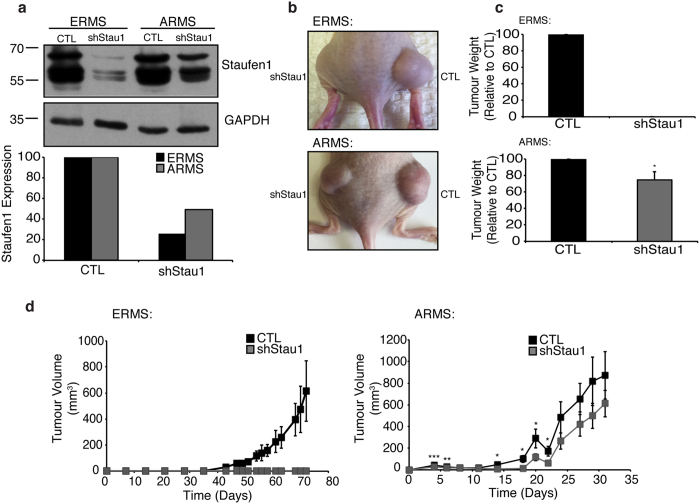
Staufen1 knockdown inhibits RMS tumour growth in a xenograft model. Flank injections of 6-week-old female SCID-488 mice with ERMS (RD) or ARMS (RH30) cells following 72 hours of Control (CTL) or Staufen1-shRNA (shStau1) expression. To determine Staufen1 knockdown pre-injection, cells were collected for western blot analysis with (**a**) anti-Staufen1 antibodies and GAPDH as a loading control in ERMS (RD) and ARMS (RH30) cells. Quantification is represented as a percentage relative to CTL (n = 1, batch culture). (**b**) Representative endpoint images of ERMS (RD) and ARMS (RH30) at 72 days and 31 days post-injection, respectively. (**c**) Tumour weight from CTL or shStau1 at endpoint for ERMS (RD) (n = 7) and ARMS (RH30) cells (n = 6). (**d**) Tumour volume measurements of ERMS (RD) and ARMS (RH30) tumours over time (n = 7 and n = 6, respectively). Data are Mean ± SEM, *P < 0.05, **P < 0.01, ***P < 0.001.

Based on the role of Staufen1 in RMS cells in culture and the increase in Staufen1 expression observed in RMS cells and tumours, we next determined whether Staufen1 knockdown impacted RMS tumour growth *in vivo*. To this end, we performed flank injections of ERMS and ARMS cells expressing CTL- or Staufen1-shRNAs. Western blotting demonstrated an ~75% and ~50% knockdown in ERMS and ARMS cells, respectively, prior to injection (Fig. 6a). Tumour growth was monitored until an ethical endpoint of 72 and 31 days for ERMS and ARMS, respectively. Strikingly, ERMS cells expressing Staufen1-shRNAs did not form tumours (Fig. 6b). The right flank injected with CTL ERMS cells formed large tumours as compared to the left flank injected with Staufen1-shRNA expressing ERMS cells (Fig. 6b,c; top panel). Although Staufen1-shRNA expressing ARMS cells formed tumours, these were significantly smaller as compared to CTL tumours (Fig. 6b; bottom panel). Total tumour weight was determined and ARMS tumours expressing Staufen1-shRNA weighed ~25% less (P < 0.05) than contralateral CTL tumours (Fig. 6c; bottom panel). Tumour volume was monitored throughout the growth period and consistent with our endpoint results, these data showed that Staufen1-shRNA expressing ERMS and ARMS tumours had decreased tumour volumes (Fig. 6d). It is important to note that in ARMS tumours, there was large variability between animals towards the end of the time-course and thus only a trend was observed from days 24–31. Analyses of Staufen1 expression in endpoint ARMS tumours demonstrated a ~30% decrease, which is less than the ~50% knockdown observed pre-injection (Figure S3a). In addition, hematoxylin and eosin staining of CTL and shStau1 tumours demonstrated that the tumours appear similar (Figure S3b). Based on these data, we hypothesize that the injected cells with significant Staufen1 knockdown were removed via apoptosis and the surviving cells formed smaller tumours consistent with those in CTL conditions. Together, these findings demonstrate that the increased expression of Staufen1 in RMS promotes tumour formation *in vivo*.

### Differential Role for Staufen1 in ERMS and ARMS

**Figure 7:**
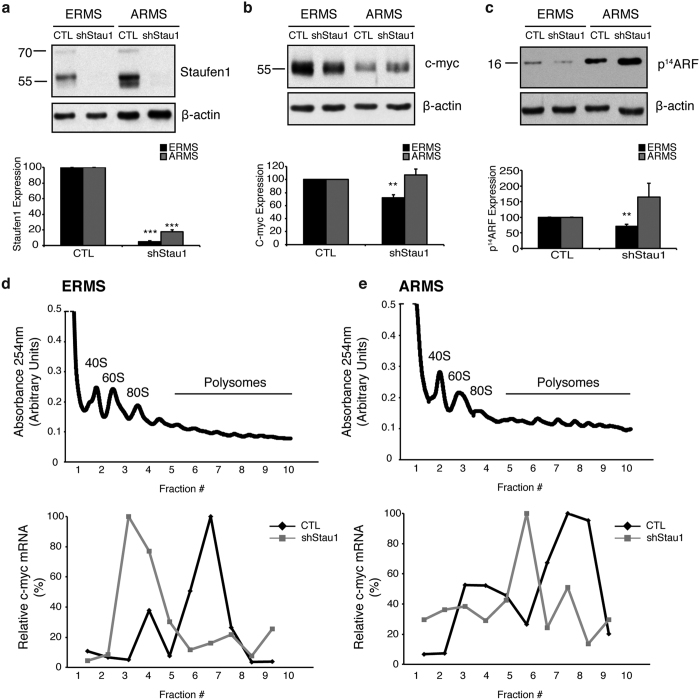
Staufen1 has differential roles in ERMS and ARMS via c-myc-dependent and -independent events, respectively. Western blot analysis of ERMS (RD) and ARMS (RH30) cells following 48 hours of Control (CTL) or Staufen1-shRNA (shStau1) expression for (**a**) Staufen1 expression (**b**) c-myc expression and (**c**) p^14^ARF. All quantifications are normalized to β-actin and represented as a percentage of the Control (CTL) with n = 3. Polysome profiling of (**d**) ERMS (RD) and (**e**) ARMS cells expressing CTL or shStau1. Polysome profile obtained by continuous absorbance readings at 254 nm (Top) and c-myc mRNA expression measured by qRT-PCR from 10 × 1 mL sucrose gradient fractions (Bottom). Data are Mean ± SEM, *P < 0.05, **P < 0.01, ***P < 0.001.

Our data demonstrate that Staufen1 has differential roles in ERMS and ARMS. Based on our previous findings that Satufen1 regulated c-myc translation^22^ and given the well-known implication of c-myc in RMS^15^,^18^,^19^,^38^, we investigated the impact of Staufen1 on c-myc expression in ERMS and ARMS. First, we evaluated c-myc expression in ERMS and ARMS cells expressing Staufen1-shRNA. Western blot analysis demonstrated an ~99% and ~95% decrease, in Staufen1 expression, respectively (Fig. 7a). We observed an ~28% reduction in c-myc protein expression in ERMS cells upon Staufen1 knockdown (P < 0.01, Fig. 7b). These data are consistent with our previous study showing a decrease in c-myc expression in C2C12 and HSMM cells expressing Staufen1-shRNA^22^. Conversely, c-myc expression was unaffected following Staufen1 knockdown in ARMS cells (Fig. 7b). Next, we analyzed expression of p14^ARF^, a downstream target of c-myc to determine if an ~28% decrease in c-myc is sufficient to impair c-myc function. Our data showed that p14^ARF^ was decreased, but only in ERMS cells with reduced Staufen1 expression (~29% decrease, P < 0.01, Fig. 7c). Interestingly, the relative expression of both c-myc and p14^ARF^ are decreased similarly in ERMS cells (Fig. 7b,c). Such a reduction was not observed in ARMS, which actually showed a trend towards increased p14^ARF^ expression (~65% increase, P = 0.1, Fig. 7c).

To further evaluate the differential roles of Staufen1 in ERMS and ARMS, we performed polysome-profiling experiments with CTL and Staufen1-shRNA expressing cells. The optical density profiles for each sucrose gradient were measured at 254 nm and profiles for ERMS and ARMS cells are depicted (Fig. 7e,d). The top of the sucrose gradient contains free mRNAs and the 40S, 60S and 80S ribosomal subunits, while mRNAs associated with polysomes are concentrated at the bottom of the gradient. Next, we examined the expression profile of c-myc mRNAs across each gradient by qRT-PCR. Our data demonstrate that c-myc mRNAs are poorly translated in ERMS cells expressing Staufen1-shRNA (Fig. 7d). These findings are entirely consistent with our previous work in C2C12 cells^22^ and support the notion that Staufen1 regulates c-myc translation in ERMS cells. In contrast, Staufen1 knockdown in ARMS cells only modestly affects c-myc mRNA localization since it remained concentrated in the polysome fractions (Fig. 7e). Together, these data highlight the impact of Staufen1 in RMS, and further demonstrate that Staufen1 regulates ERMS and ARMS tumorigenesis via c-myc-dependent and -independent events.

## Discussion

Given the well documented impact of c-myc in RMS tumorigenicity^15^,^16^,^17^,^18^,^19^,^20^,^38^, and our recent findings showing that Staufen1 regulates c-myc during skeletal muscle differentiation^22^, we hypothesized in the current study that Staufen1 controls RMS tumorigenicity. We thus performed a series of complementary experiments to examine whether Staufen1 controls c-myc expression in two RMS subtypes, and determined the impact of this regulatory pathway on tumorigenesis. Results showed that Staufen1 is overexpressed in ERMS and ARMS tumours and cell lines. Similarly, c-myc protein but not mRNA was also increased in both subtypes. Moreover, Staufen1 specifically regulates the proliferation and apoptosis of ERMS and ARMS cells, respectively, thereby highlighting the differential roles of Staufen1 in these RMS subtypes. Finally, knockdown of Staufen1 decreased invasion and migration in ERMS and ARMS cultures, while also inhibiting tumour growth *in vivo*. Together, our data provide the first direct evidence for the impact of Staufen1 in cancer biology.

Converging lines of evidence indicate that Staufen1 plays an important regulatory role during skeletal muscle differentiation as well as in neuromuscular disorders^22^,^23^,^24^,^25^,^26^,^28^,^29^,^48^,^49^. In particular, we showed that Staufen1 is down-regulated during myogenesis and embryonic muscle development, but that its expression remains preferentially high within the post-synaptic sarcoplasm of neuromuscular junctions^22^,^24^. Moreover, sustained expression of Staufen1 causes myoblasts to differentiate poorly as evidenced by inhibition of fusion and a decrease in several differentiation markers including MyoD, myogenin, MEF2A and MEF2C^22^. Similarly, Yamaguchi *et al*. reported that Staufen1 negatively regulates muscle differentiation^48^ via regulation of Dvl2 mRNAs^49^. In addition, it has also been suggested that Staufen1 controls myogenesis through a process referred to as Staufen1-mediated decay (SMD)^22^,^28^,^29^. Taken together, results from these studies clearly highlight the role of Staufen1 during myogenesis.

In our recent work, we uncovered that Staufen1 increases expression of c-myc through a translational mechanism that involves its 5′UTR. An increase of c-myc, in turn, delays cell cycle exit thereby causing impaired differentiation^22^. Such regulation of c-myc translation by Staufen1 is consistent with the ability of Staufen1 to promote translation of target mRNAs via secondary structures formed within 5′UTRs^31^. Along those lines, more recent work further showed that Staufen1 can regulate translation via secondary structures located throughout mature transcripts^32^,^33^. Of relevance, a similar role for Staufen1 in regulating c-myc expression was reported in a study focused on IGF2BP1 in osteosarcoma U2OS cells^50^. In the latter study, Staufen-containing mRNPs co-purified with IGF2BP1-containing mRNPs, and Staufen1 knockdown decreased c-myc protein expression without parallel alterations in mRNA levels^50^. Our current results obtained with ERMS cells are thus entirely consistent with these earlier studies showing that Staufen1 exerts a profound translational regulatory influence on c-myc mRNAs. The importance of translational regulation has been described for other RBPs such as Y-box binding protein 1 (YB-1), which increases translation of specific target mRNAs causing, for example, enhanced metastatic potential of breast cancer cells^51^ and drives sarcoma invasion and metastasis^52^. In the context of RMS, it was recently shown that IGF2BP1 regulates translation of mRNAs encoding cellular inhibitor of apoptosis 1 (cIAP1) thus impacting ERMS cell survival and drug resistance^53^.

The role of c-myc in driving oncogenesis has been extensively studied for several years^54^. More specifically, the reduction of c-myc through the Ras/MEK/ERK signaling greatly reduced ERMS malignancy and radioresistance^15^,^16^,^17^,^18^,^19^,^20^,^38^. Although c-myc protein expression is increased in both ERMS and ARMS cell lines and primary tumour samples, it appears that Staufen1 has different roles in regulating its expression. In ERMS cells, Staufen1-mediated regulation of c-myc is consistent with the expected translational regulation as discussed above and the decrease in proliferation we observed. By contrast, knockdown of Staufen1 in ARMS cells is unable to modulate c-myc expression. However, reduction of Staufen1 in ARMS increased apoptosis of ARMS cells in culture, without affecting proliferation, and also inhibited tumour growth in a xenograft model. A similar effect of Staufen1 knockdown was observed in neuronal cells since disruption of any component of the TDP-43/FMRP/Staufen1 complex, including with a siRNA against Staufen1, sensitized these cells to apoptosis^55^. Combined with our findings, these data indicate that in addition to Staufen1’s role in controlling exit/entry of the cell cycle^22^,^34^, Staufen1 can also regulate apoptosis in specific cellular contexts. Altogether, these data not only highlight the differential roles of Staufen1 in RMS, but also strengthen the importance of Staufen1-regulated c-myc events.

Through activation of p14^ARF^, c-myc modulates the activity of the tumour suppressor protein p53, inducing apoptosis^56^. Although the ERMS RD cell line used contains a homozygous mutation in the *TP53* gene, rendering it non-functional, the ARMS RH30 cell line contains a heterozygous mutation leaving one functional *TP53* allele^41^,^57^. In the current study, the knockdown of Staufen1 failed to regulate c-myc expression in ARMS cells. Given the increased p14^ARF^ expression, it seems that this may be sufficient to activate p53 and increase apoptosis in ARMS cells. Therefore, sustained c-myc expression and increased p14^ARF^ in ARMS, despite the Staufen1 knockdown, likely contributes to the increased apoptosis observed in these cells.

In recent years, Staufen1 has emerged as a multi-functional RBP involved in several key aspects of RNA metabolism including mRNA localization^27^, stability^28^,^29^,^30^, translation^22^,^31^,^32^,^33^, and alternative splicing^23^,^25^,^33^. Therefore, it seems most likely that Staufen1 regulates other target mRNAs in ARMS, which act in combination with c-myc regulated p53-dependent apoptosis, to amplify the apoptotic response. In this context, several groups have performed large scale screens to identify Staufen1-interacting proteins and mRNA binding sites across various cell types, adding to the complexity of Staufen1-regulated events^32^,^33^,^58^,^59^. In addition, small and large-scale screens have also been performed on ARMS cells and tumours to better understand the impact of the PAX3- or PAX7-FOXO1 fusion proteins^60^,^61^,^62^,^63^,^64^,^65^,^66^. Comparative analysis of Staufen1-regulated mRNAs with the disrupted genes and molecular pathways caused by the oncogenic fusion proteins may identify potential Staufen1 targets relevant for ARMS. For example, *FGFR1, KRAS, NRAS, MDM2*, and *CDK4* are commonly misregulated in fusion-positive ARMS^44^,^62^ and, interestingly, each contains at least one Staufen1-binding site in their coding sequence (CDS) or 3′UTR^33^. The identification of all Staufen1 targets will prove invaluable for comprehensively deciphering its regulatory function in fusion-positive ARMS.

Recent work from DesGroseillers and colleagues demonstrated the cell cycle-dependent regulation of Staufen1 expression in various cancerous cell lines (HCT116, U2OS, and HEK293T). Based on these observations, the authors hypothesized that Staufen1 may participate in the cell cycle progression of cancer cells^34^. Our recent study describing the impact of Staufen1-dependent regulation of c-myc expression in skeletal muscle also indicated that Staufen1 is involved in controlling the proliferative capacity of cells^22^. Here, using multiple approaches, we thus provide direct evidence that Staufen1 regulates key tumorigenic features of cancer cells both *in vitro* and *in vivo* thereby revealing for the first time its crucial involvement in cancer biology. Such a novel function of Staufen1 in RMS clearly highlights its potential as a new and relevant target for the development of therapeutic strategies based on Staufen1 modulation that are much needed for this devastating pediatric cancer.

## Methods

### Constructs and Antibodies

The constructs used were pLKO.1-TRC cloning vector, a gift from David Root (Addgene #10878), pLKO.1-TRC-shStau1 (Clone ID: TRCN0000102306, Clone ID: TRCN0000102308 and Clone ID: TRCN0000102309) (GE Healthcare Life Sciences, Ontario, Canada), pMD2.G (Addgene #12259) and psPAX2 (Addgene #12260) were gifts from Didier Trono.

The antibodies used were anti-Staufen1 (ab73478, Abcam, Ontario, Canada), anti-c-myc (ab11917, Abcam, Ontario, Canada), anti-p^14^ARF/CDKN2A (NB200-111, Novus Biologicals, Ontario, Canada), anti-myosin heavy chain (MF20, Developmental Studies Hybridoma Bank, Iowa City, IA) anti-β-actin (#47778, Santa Cruz Biotechnology, CA, USA), anti-Tubulin (T5168, Sigma-Aldrich, Ontario, Canada) and anti-GAPDH (ab8245, Abcam, Ontario, Canada).

### Cell Culture, Transfection, and Lentivirus Production and Infection

Clonetics Skeletal Muscle Cells (SkMC) and Human Skeletal Muscle Myoblasts (HSMM) were cultured in according to manufacturer instructions (Lonza, NJ, USA). Embryonal rhabdomyosarcoma RD cells (CCL-136) and HEK 293T cells (CRL-3216) were cultured according to manufacturer instructions (American Type Culture Collection, VA, USA). Alveolar rhabdomyosarcoma RH30 cells (CRL-2061; American Type Culture Collection, VA, USA) and the RH36, RH18 and RH41 cells, which were a gift from Dr. P. Houghton (Department of University of Hematology-Oncology, St Jude Children’s Research Hospital), were cultured in Multicell RPMI 1640 1X with L-Glutamine (Wisent Bioproducts, Quebec, Canada) supplemented with 15% HyClone FBS and 1% HyClone Penicillin-Streptomycin (Thermo Fisher Scientific, Ontario, Canada). All cell cultures were incubated at 37 °C, 5% CO_2_.

Lentiviral particles were produced in HEK-293T cells by transfection of CTL or shRNA vectors and packaging vectors psPAX2 and pMD2.G using Lipofectamine 2000 (Invitrogen, Ontario, Canada) according to the manufacturers protocol. Medium containing viral particles was collected by centrifugation (1000 rpm, 5 min) and filtered (0.45 μm). Cells were infected twice with equal volumes of virus and media containing 8 μg/ml Hexadimethrine Bromide (Sigma-Aldrich, Ontario, Canada). Cells were collected 48–72 h post-secondary infection.

### Western Blotting

Cells were lysed in RIPA buffer as previously described^22^, sonicated and centrifuged (13000 rpm, 10 min). Protein concentration was determined with the Bicinchoninic Acid protein assay kit (Thermo Fisher Scientific, Ontario, Canada) and 10–30 μg of protein was separated by SDS-PAGE and transferred onto nitrocellulose membranes (Bio-Rad, Ontario, Canada). Membranes were then blocked and incubated with antibodies as previously described^22^. For endogenous Staufen1 expression in RMS cells, only the predominant Stau1-55 isoform was quantified whereas total Staufen1 expression was quantified following knockdown.

### RNA Extraction, Reverse Transcription, and Real-Time quantitative PCR

RNA extraction, reverse transcription and qRT-PCR were performed as previously described^22^. Primer sequences were as follows: Staufen1 (fwd 5′-AACGGAACTTGCCTGTGAAT-3′, rev 5′-AGGGGCGGTAACTTCTTCAG-3′), c-myc (fwd 5′-CCTACCCTCTCAACGACAGC-3′, rev 5′-CTCTGACCTTTTGCCAGGAG-3′) and 18S (fwd 5′- GTAACCCGTTGAACCCCATT-3′, rev 5′-CCATCCAATCGGTAGTAGCG-3′).

### Human Samples

Frozen and formalin-fixed, paraffin-embedded human pediatric RMS tumours and normal skeletal muscle samples were obtained from either the Children’s Hospital of Eastern Ontario (CHEO) or from the Ontario Tumour Bank after institutional ethics board approval from CHEO and The Ottawa Hospital, respectively. Biological materials were provided by the Ontario Tumour Bank, which is funded by the Ontario Institute for Cancer Research. All primary tumour samples were collected following informed consent and all experiments were performed in accordance with relevant guidelines and regulations. Control human muscle lysates were purchased (Novus Biologicals, Ontario, Canada). TMA slides were purchased from US Biomax, Inc. (Rockville, MD, USA).

### Immunohistochemistry and Immunofluorescence

Formalin-fixed, paraffin-embedded TMA slides were deparaffinized in xylene and rehydrated through a 100–70% ethanol gradient. Heat induced epitope retrieval was done at 110 °C, 12 min with citrate buffer pH 6.0 and 3% H_2_O_2_ was used to block endogenous peroxidases. Sections were blocked with Background Sniper blocking reagent (BioCare Medical, CA, USA) and then incubated with anti-Staufen1 antibodies for 1 h at room temperature (1:500 antibody dilution). The MACH4^TM^ + DAB detection system was used according to manufacturers instruction (BioCare Medical, CA, USA). Nuclei were counter-stained using Hematoxylin. A pathologist analyzed and scored the sections for staining intensity and a 4-point scale was developed to score Staufen1 staining in the tissues, where 0–1 is low, 2–3 is moderate, and 4 represents high Staufen1 expression. In most cases, the percentage of cells positive for Staufen1 staining was ~90–100%. Only in four cases was the percentage of positive cells below ~20% and these tumours were scored as 0–1 staining intensity since these tumour cells had a high nuclear/cytoplasmic ratio, making it difficult to analyze with light microscopy. Therefore, the assessment of percentage of positive cells was deemed to be non-contributory.

Xenograft tumours were dissected and paraffin-embedded. Sections (10 μm) were stained with Hematoxylin and Eosin followed by a series of dehydration steps via ethanol washes (70%, 95%, 100%), sections were cleared with toluene and mounted using Permount (Fisher Scientific, Ottawa, Canada). Stained samples were then visualized by light microscopy under 20X magnification.

For immunofluorescent staining, cells were fixed for with 3% formaldehyde in 1X PBS pH 7.4 for 30 min and then permeablized with 0.5% Triton X-100 in 1X PBS pH 7.4 for 15 min. Cells were then blocked with 10% fetal bovine serum (FBS) for 1 h at room temperature. Cells were incubated with the primary antibody diluted in 1% FBS, 0.1% Triton X-100 overnight at 4 °C. Next the cells were washed 3 × 5 min with 1X PBS pH 7.4 and then incubated with Alexa secondary antibodies (Invitrogen, Ontario, Canada) for 1–2 h at room temperature. Cells were washed 3 × 5 min with 1X PBS pH 7.4 and then mounted on slides using Vectashield mounting media containing DAPI for staining nuclei (Vector Labs, Ontario, Canada). Cells were visualized by microscopy using a Zeiss AxioImager.M2 microscope.

### Proliferation Assay and Cell Cycle Analysis

Cell proliferation was quantified by flow cytometry using 5-bromo-2′-deoxyuridine (BrdU) incorporation. Cells were seeded on 60 mm culture plates (7.5 × 10^5^), infected with CTL or Staufen1-shRNA lentivirus and maintained at 70% confluency. BrdU (30 μM) (Invitrogen, Ontario, Canada) was added to cultures 72 h post-secondary infection and incubated for 2 h. Labeled cells were washed with sterile PBS and collected using HyClone Trypsin 0.25% (1X) solution. Cells were washed and fixed with 5 mL of cold 70% ethanol for 30 min at −20 °C. Cells were centrifuged (1200 rpm, 7 min), washed and collected by centrifugation (1200 rpm, 7 min). Cells were resuspended in 1 mL of sterile PBS containing 0.5 mg/mL RNase A (QIAGEN, Ontario, Canada) and incubated for 30 min at 37 °C, then washed with 1 mL of sterile PBS and centrifuged (1200 rpm, 7 min). Samples were resuspended in 1 mL of cold 0.1 N HCl (Sigma-Aldrich, Ontario, Canada) containing 0.7% Triton X-100 (Sigma-Aldrich, Ontario, Canada) and incubated at 4 °C for 15 min. Subsequent washing with 1 mL of sterile PBS, centrifugation (1200 rpm, 7 min) and resuspension in 1 mL of sterile H_2_O, incubated at 97 °C for 15 min and then 4 °C for 15 min. Next, cells were resuspended in 1 mL of 0.5% Tween 20 (Sigma-Aldrich, Ontario, Canada) and were collected by centrifugation (1200 rpm, 7 min). Pellets were suspended in 100 μl of HBT (0.5% Tween 20, 5% w/v BSA in sterile PBS) containing Alexa Fluor^®^ 488 anti-BrdU antibody (1:20) (Invitrogen, Ontario, Canada) and incubated at room temperature for 30 min. 1 mL of HBT was added and cells were centrifuged (1200 rpm, 7 min). Finally, cells were suspended in 0.5 mL of 20 μg/mL PI (Sigma-Aldrich, Ontario, Canada) and 100 μg/mL RNase A (QIAGEN, Ontario, Canada), transferred to 5 mL Falcon^®^ polystyrene round bottom tubes (Thermo Fisher Scientific, Ontario, Canada) and incubated at 4 °C for 30 min. Stained cells were analyzed using the Beckman MoFlo^®^ Astrios^TM^ or the BD LSRFortessa^TM^ flow cytometers. Data analysis was performed using FlowJo software. Cell cycle was analyzed using the Dean-Jett-Fox model.

### Apoptosis Assay

Cells were seeded in 60 mm culture plates (7.5 × 10^5^), infected with CTL or Staufen1-shRNA lentivirus and maintained at 70% confluency. Cells were co-stained 72 h post-secondary infection using the Alexa Fluor^®^ 488 Annexin V/Dead Cell Apoptosis Kit with Alexa Fluor^®^ 488 annexin V and PI for Flow Cytometry (Thermo Fisher Scientific, Ontario, Canada) according to manufacturer instructions. Stained cells were analyzed using the Beckman MoFlo^®^ Astrios^TM^ or the BD LSRFortessa^TM^ flow cytometers. Data analysis was performed using FlowJo software.

### Motility and Invasion Assay

Infected cells (2.5 × 10^4^) were seeded in serum free medium in Corning^®^ BioCoat^TM^ Control Inserts (#354578) or Corning^®^ GFR Matrigel^®^ Basement Membrane Matrix Invasion Chambers (#354480) (VWR International, Ontario, Canada) containing growth medium in the bottom chamber. RD and RH30 cells were incubated for 72 and 48 h, respectively. Cells were fixed and stained with the Shandon^TM^ Kwik-Diff^TM^ Stain (Thermo Fisher Scientific, Ontario, Canada). Cell motility and invasion were assessed according to manufacturer instructions. Six random fields of view were imaged and analyzed using Northern Eclipse Software (NES, Expix Imaging, Ontario, Canada).

### Migration Assay

RD and RH30 cells were infected with CTL or Staufen1-shRNA lentivirus for 48 h post-secondary infection and were seeded into culture inserts (RD CTL 7.5 × 10^5 ^cells/ml; RD shStau1 1 × 10^6 ^cells/ml; RH30 CTL; shStau1 5 × 10^5 ^cells/ml) to form a confluent monolayer (Ibidi, Munich, Germany). Inserts were removed 24 h post-seeding and washed with PBS and imaged in real time for 30 h using the Incucyte^®^ ZOOM system (Essen Bioscience, MI, USA). The gap was measured using Northern Eclipse Software (NES, Expix Imaging, Ontario, Canada). The average distance at 3 points was calculated. These values were normalized to the % confluence of cells.

### Xenograft Mouse Model

Animal experimental protocols were approved by the University of Ottawa Animal Care Committee and were in accordance with the Canadian Council of Animal Care Guidelines. RD and RH30 cells were infected with lentivirus encoding CTL or Staufen1-shRNA and collected 72 h post-secondary infection. Subcutaneous tumours were established by performing flank injections with 2.5 × 10^6^ and 5 × 10^6^ RD and RH30 cells, respectively, on randomly selected 6-week-old female SCID hairless mice SHC strain 488 (Charles River, Saint-Constant, Canada). The right flank injection contained pLKO.1-TRC-Control expressing cells whereas the left flank injection contained pLKO.1-Staufen1-shRNA expressing cells. Tumour width and length were measured throughout experiment (unblinded) and tumour volume was calculated using [(L*W^2^)/2] where L is the tumour length and W is the tumour width. One animal was excluded as no tumours formed during the experimental timeline. Each mouse represented a biological replicate, and both littermates and non-littermates were used.

### Polysome Profiling

RD and RH30 cells were infected with two rounds of CTL or Staufen1-shRNA and were grown at 50–60% confluency on 150 mm culture plates. Two plates per condition were treated at 48 h post-secondary infection with 0.1 mg/ml Cycloheximide (CHX) (Sigma-Aldrich, Ontario, Canada) for 5 min in DMEM. Cells were washed twice with ice cold 1X PBS pH 7.4 containing 0.1 mg/ml CHX, collected by centrifugation (1000 rpm × 5 min 4 °C) and resuspended in 500 uL RNA lysis buffer (0.3 M NaCl, 15 mM MgCl_2_, 15 mM Tris pH 7.4, 1% Triton X-100, 1 mM Dithiothreitol, 0.1 mg/mL CHX, 100 U/mL RNasin). Cells were incubated on ice for 10 min and lysed using a 20 gauge needle. Nuclear debris was removed by centrifugation at 5000 rpm × 10 min at 4 °C followed by a subsequent centrifugation at 13 000 rpm for 10 min at 4 °C to remove cellular debris. Next, 500 uL of cell lysate was layered on the top of continuous sucrose gradients (15–45% sucrose in 0.3 M NaCl, 15 mM MgCl_2_, 15 mM Tris pH 7.4). Ultracentrifugation of samples was performed at 39 000 rpm in a SW41-Ti rotor for 90 min at 4 °C with no brake. We collected 1 mL fractions using the BRANDEL Density Fractionation System. Samples were digested with proteinase K and total RNA was extracted and analyzed by qRT-PCR.

### Statistical Analysis

All experiments were performed with a minimum of n ≥ 3 biological replicates unless otherwise stated. Immunohistochemical data was analyzed using the Chi-Squared test with significance set at P ≤ 0.05. The data were analyzed using the student’s t-test. Significance was set at P ≤ 0.05 with *P ≤ 0.05, **P ≤ 0.01, and ***P ≤ 0.001. Error bars represent standard error of the mean (SEM).

## Additional Information

**How to cite this article:** Crawford Parks, T.E. *et al*. Novel Roles for Staufen1 in Embryonal and Alveolar Rhabdomyosarcoma via c-myc-dependent and -independent events. *Sci. Rep.* **7**, 42342; doi: 10.1038/srep42342 (2017).

**Publisher's note:** Springer Nature remains neutral with regard to jurisdictional claims in published maps and institutional affiliations.

## Supplementary Material

Supplementary Information

## Figures and Tables

**Table 1:** Summary of Staufen1 score intensity distribution.

Score	Normal Tissue (n = 8)	Embryonal RMS (n = 26)	Alveolar RMS (n = 24)
0–1 (Low)	7 (87.5%)	12 (46.2%)	16 (66.7%)
2–3 (Moderate)	1 (12.5%)	9 (34.6%)	8 (33.3%)
4+ (High)	0 (0%)	5 (19.2%)	0 (0%)
P-values		0.11	0.26

Note: P-values calculated by Chi-Squared Test.
