# Supplementary material for: Novel Roles for Staufen1 in Embryonal and Alveolar Rhabdomyosarcoma via c-myc-dependent and -independent events
Source: Sci Rep. 2017 Feb 17;7:42342. doi: 10.1038/srep42342 (PMC5314364; doi:10.1038/srep42342)
Supplement: Supplementary Information [file srep42342-s1.pdf]

Novel Roles for Staufen1 in Embryonal and Alveolar Rhabdomyosarcoma via c-myc-dependent and -independent events

Tara E. Crawford Parks[1,2]; Kristen A. Marcellus[1,2]; Jonathan Langill[1,2]; Aymeric Ravel-Chapuis[1,2]; Jean Michaud[2,3]; Kyle N. Cowan[1,4,5]; Jocelyn Côté[1,2]; Bernard J. Jasmin[1,2]*.

Supplementary Information

Figure Legends

Figure S1. Staufen1 and c-myc are increased in human primary ERMS and ARMS tumours and multiple RMS cell lines. **(a)** Analysis of Staufen1 expression and **(b)** c-myc expression by western blot in ERMS (n=4) and ARMS (n=1) human primary tumour samples, normal fetal (n=1) and adult skeletal muscle (n=1) tissues with GAPDH as a loading control. Quantifications are represented as a percentage relative to adult skeletal muscle. **(c)** Analysis of Staufen1 and **(d)** c-myc expression by western blot in ERMS cells (RD, RH36, and RH18) and ARMS cells (RH30, RH41) as compared to control Human Skeletal Muscle Myoblasts (HSMM), (n=2 for all conditions). The Pearson correlation coefficient for Staufen1 and c-myc is $r=0.96$, $P<0.01$, indicating a very strong correlation. Dotted lines represent expression in HSMM cells. Quantifications are represented as a Fold change relative to HSMM cells.

Figure S2. Lentiviral mediated Staufen1 knockdown in ERMS and ARMS. Immunofluorescence of ERMS (RD) and ARMS (RH30) cells after 72 h of infection with Control (CTL) or Staufen1-shRNA (shStau1) lentiviral particles. Cells were stained with an anti-Staufen1 antibody (green) and nuclei with DAPI (blue), scale bars = 50 µm.

Figure S3. Staufen1 expression in end-point ARMS xenograft tumours. Control (CTL) and Staufen1-shRNA (shStau1) expressing ARMS tumours were dissected and paraffin-embedded at day 31 post-injection. (**a**) Staufen1 expression in end-point ARMS tumours was examined by western blot using anti-Staufen1 antibodies and anti-GAPDH antibodies as a loading control. Quantification is represented as a percentage relative to CTL (n=6) and data are Mean ± SEM. (**b**) Representative sections of CTL and shStau1 ARMS tumours stained with Hematoxylin and Eosin, scale bar = 50 μm.

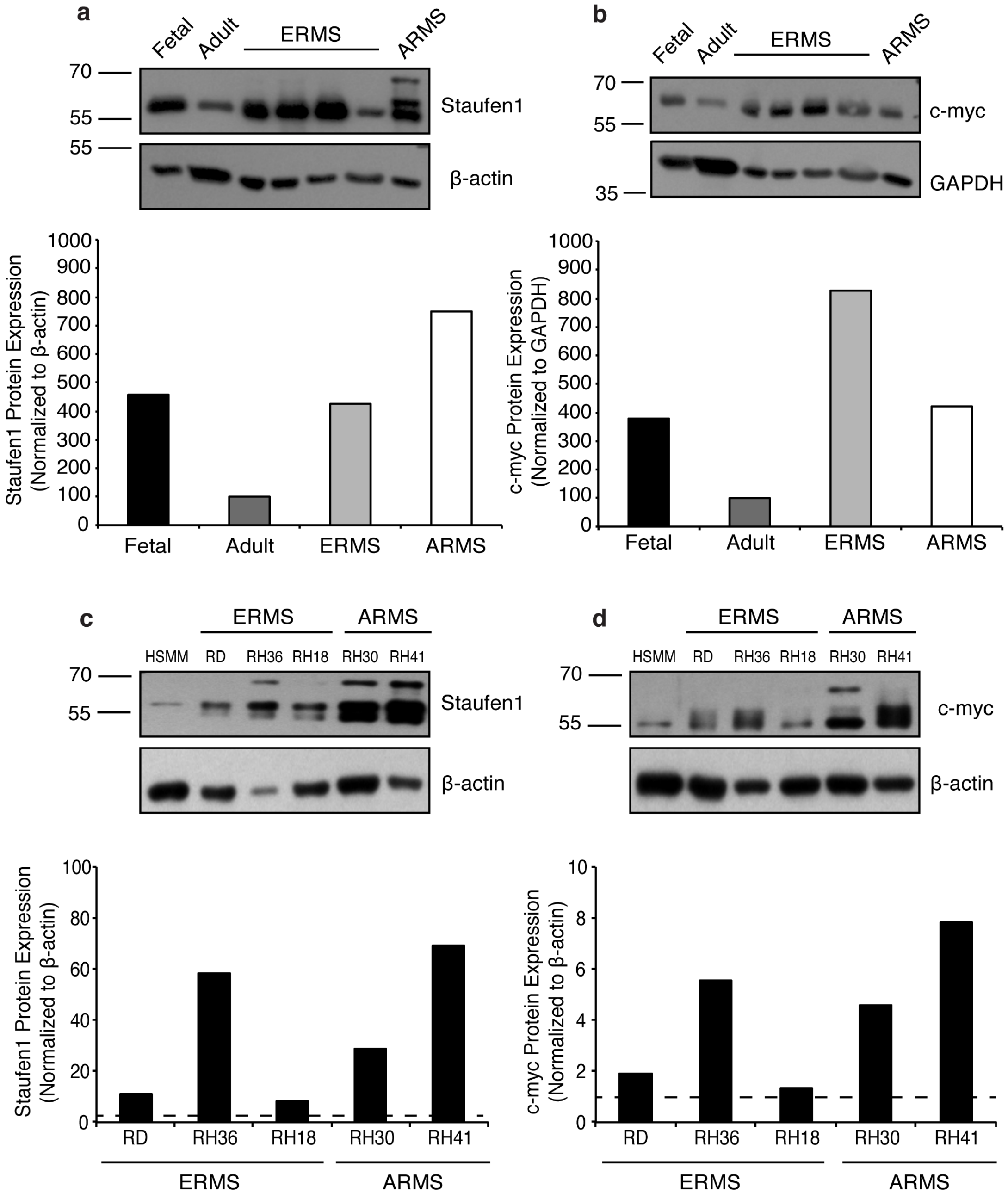
a
Fetal
Adult
ERMS
ARMS
70
55
55
Staufen1
β-actin
Staufen1 Protein Expression
(Normalized to β-actin)
1000
900
800
700
600
500
400
300
200
100
0
Fetal
Adult
ERMS
ARMS
b
Fetal
Adult
ERMS
ARMS
70
55
35
c-myc
GAPDH
c-myc Protein Expression
(Normalized to GAPDH)
1000
900
800
700
600
500
400
300
200
100
0
Fetal
Adult
ERMS
ARMS
c
ERMS
ARMS
HSMM
RD
RH36
RH18
RH30
RH41
70
55
Staufen1
β-actin
Staufen1 Protein Expression
(Normalized to β-actin)
100
80
60
40
20
0
RD
RH36
RH18
RH30
RH41
ERMS
ARMS
d
ERMS
ARMS
HSMM
RD
RH36
RH18
RH30
RH41
70
55
c-myc
β-actin
c-myc Protein Expression
(Normalized to β-actin)
10
8
6
4
2
0
RD
RH36
RH18
RH30
RH41
ERMS
ARMS

Figure S1

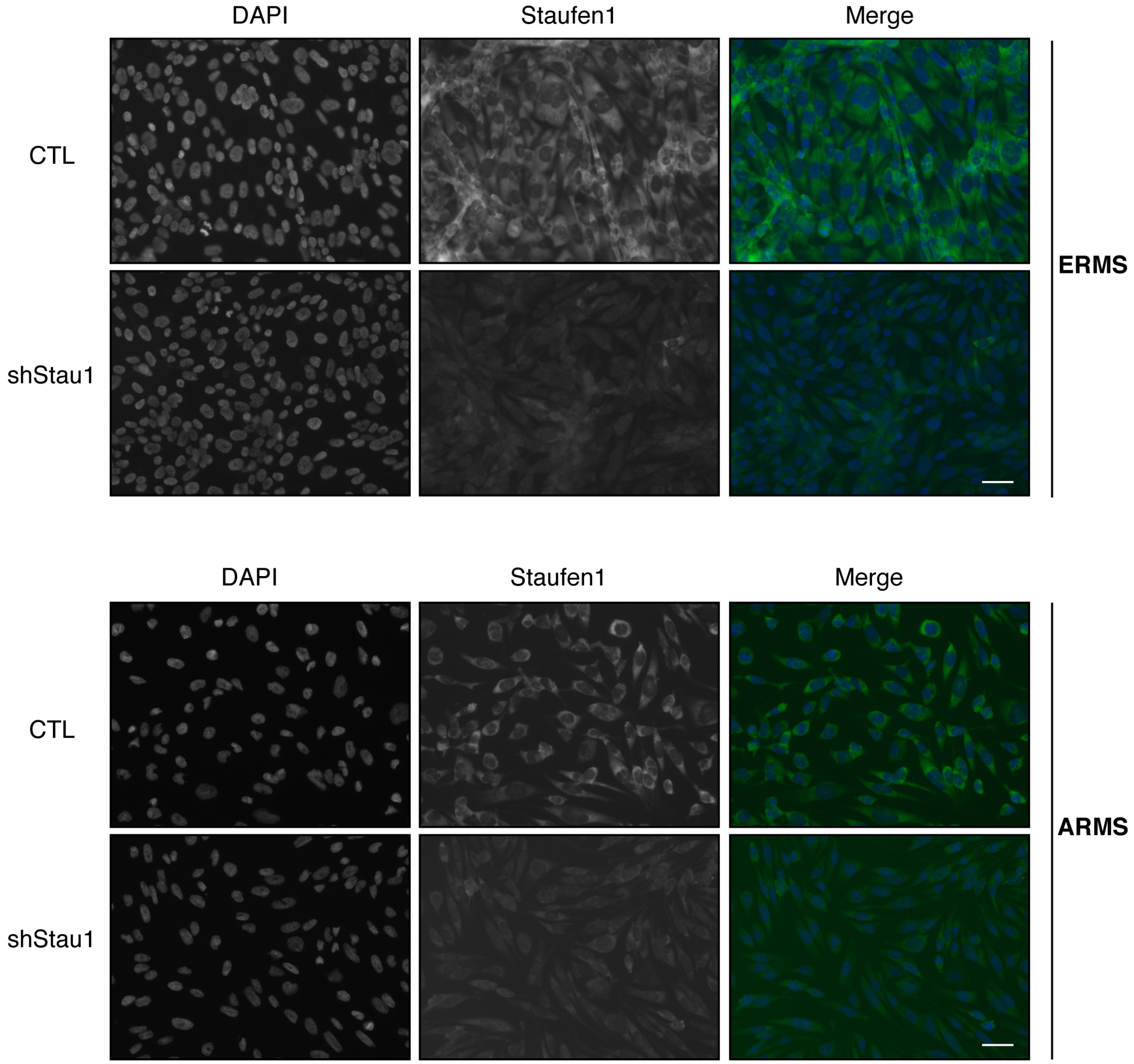
DAPI
Staufen1
Merge
CTL
shStau1
ERMS
DAPI
Staufen1
Merge
CTL
shStau1
ARMS

34 Figure S2

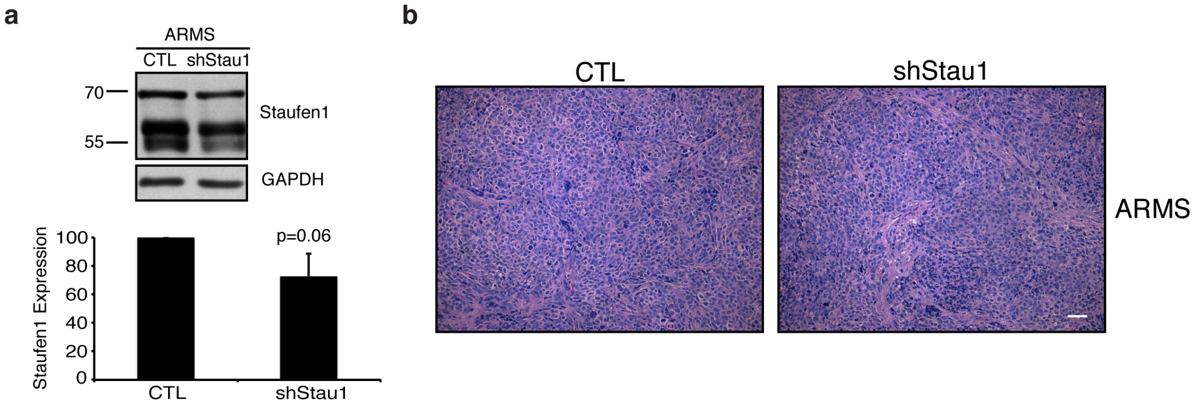
a
ARMS
CTL
shStau1
70
55
Staufen1
GAPDH
Staufen1 Expression
100
80
60
40
20
0
p=0.06
CTL
shStau1
b
CTL
shStau1
ARMS

Figure S3
